# Comparison of transcriptional responses in liver tissue and primary hepatocyte cell cultures after exposure to hexahydro-1, 3, 5-trinitro-1, 3, 5-triazine

**DOI:** 10.1186/1471-2105-7-S4-S22

**Published:** 2006-12-12

**Authors:** Edward J Perkins, Wenjun Bao, Xin Guan, Choo-Yaw Ang, Russell D Wolfinger, Tzu-Ming Chu, Sharon A Meyer, Laura S Inouye

**Affiliations:** 1 US Army Engineer Research and Development Center, 3909 Halls Ferry Road, Vicksburg, MS, USA; 2 SAS Institute Inc, SAS Campus Drive, Cary, NC, USA; 3 SpecPro, Vicksburg, MS, USA; 4 University of Louisiana at Monroe, Monroe, LA, USA

## Abstract

**Background:**

Cell culture systems are useful in studying toxicological effects of chemicals such as Hexahydro-1,3,5-trinitro-1,3,5-triazine (RDX), however little is known as to how accurately isolated cells reflect responses of intact organs. In this work, we compare transcriptional responses in livers of Sprague-Dawley rats and primary hepatocyte cells after exposure to RDX to determine how faithfully the *in vitro *model system reflects *in vivo *responses.

**Results:**

Expression patterns were found to be markedly different between liver tissue and primary cell cultures before exposure to RDX. Liver gene expression was enriched in processes important in toxicology such as metabolism of amino acids, lipids, aromatic compounds, and drugs when compared to cells. Transcriptional responses in cells exposed to 7.5, 15, or 30 mg/L RDX for 24 and 48 hours were different from those of livers isolated from rats 24 hours after exposure to 12, 24, or 48 mg/Kg RDX. Most of the differentially expressed genes identified across conditions and treatments could be attributed to differences between cells and tissue. Some similarity was observed in RDX effects on gene expression between tissue and cells, but also significant differences that appear to reflect the state of the cell or tissue examined.

**Conclusion:**

Liver tissue and primary cells express different suites of genes that suggest they have fundamental differences in their cell physiology. Expression effects related to RDX exposure in cells reflected a fraction of liver responses indicating that care must be taken in extrapolating from primary cells to whole animal organ toxicity effects.

## Background

RDX is known to contaminate soil and ground water associated with munitions manufacturing and artillery training sites [1], some of which are listed on the National Priorities List by the U.S. Environmental Protection Agency [2]. RDX has also been targeted for future regulatory action under provisions of the Safe Drinking Water Act [3]. Human health effects of RDX are well known to include the central nervous system as the critical target tissue for acute exposure to high doses as manifested by reversible seizure activity [2,4-6]. While many effects of RDX exposure are known, little is known about the mechanisms by which it causes toxicity.

Primary cell cultures offer several advantages over animals in determining mechanisms of chemical toxicity. Primary hepatocyte cells are freshly prepared from liver tissue by proteolytic digestion and enrichment of hepatocyte cells through centrifugation. These cells enable high throughput testing methods to examine toxicity. Primary cell cultures can reduce concerns such as animal availability, cost, and welfare that limit the application of *in vivo *studies of chemical effects. However, the responses to chemical exposure in isolated cells may not reflect those of intact organs in animals especially as organs are often composed of more than one cell type potentially complicating direct comparisons. Liver cell populations consist of approximately 85% hepatocytes along with other cell types: Kupfer (macrophage) cells, vascular endothelial cells and "Ito" cells (a myeloepithelial cell) [7]. In addition, conservation of gene expression patterns in hepatocytes isolated from livers have been shown to diverge from liver tissue as the cells adapt to culture conditions [8,9] and may respond to chemicals in a manner different from liver tissue [10].

We examined *in vitro *exposed primary hepatocyte cells and *in vivo *exposed rat liver tissue to understand how the transcriptional responses of these systems compared after exposure to RDX. Gene expression was analyzed in primary cell cultures exposed to 7.5, 15, or 30 mg/L RDX for 24 and 48 hours. Primary cell expression effects were compared to those in liver tissue isolated from female Sprague-Dawley rats 24 hours after gavage with 12, 24, or 48 mg/Kg RDX. Samples were assessed within time point and cell type (24 hr cell, 48 hr cell, and liver tissue) to their respective controls using a loop design and 2-color, 8 K gene microarrays.

## Results

We have examined the gene expression responses in primary hepatic cells freshly isolated from liver tissue. Exposure concentrations for transcriptional profiling of cells were selected based on the results of cytotoxicity assays, which indicated a complete lack of cytotoxicity at all levels of RDX tested. Concentrations to which cells were exposed were similar to the 8.4 mg/kg tissue found in liver 24 hrs after dosing with 47 mg/Kg RDX (data not shown) given the assumption that tissue is approximately 80% water and 1 gm/ml resulting in concentration of approximately 42 mg/L water.

### Comparison of expression in unexposed cells and tissue

Gene expression was assessed using an oligonucleotide microarray capable of detecting 7616 different rat genes. Twenty five percent of all genes on the array were detected in control liver tissue, 48% of all genes in 24 hr control primary cells, and 66% of all genes in 48 hr control primary cells. Analysis of GO biological process terms associated with genes expressed in controls indicated significant differences both in the variety and type of biological processes present in cells and tissues prior to chemical exposure. Liver tissues were associated with 305 different terms with terms related to amino acid metabolism and catabolism, lipid and fatty acid metabolism, and aromatic and drug metabolism being unique to liver cells. 24 hr cells were associated with 398 different terms with terms related to cell division, organ development, response to DNA damage unique to 24 hr cells. Forty eight hr cells were associated with 587 GO biological process terms with terms related to cell cycling, protein modification, alkene metabolism, neural cell development, signalling, and regulation in addition to organ and cell differentiation, organization, and development. Comparison of expressed genes in tissues and cells versus the list of those on an array can also reveal functions that are enriched in each particular cells type (Table 1). Relative to cells, liver tissues were significantly enriched in lipid, fatty acid and steroid metabolism, electron transport, oxidative phosphorylation, and cell adhesion-mediated signalling. Twenty four hr cells were significantly enriched in protein folding, vesicle transport, protein traffic, and nucleic acid metabolism when compared to 48 hr cells and liver tissue. Forty eight hr cells were enriched for genes involved in proteolysis and circulation in comparison to 24 hr cells and liver tissue.

### Comparison of expression in RDX exposed cells and tissue

The overall response of different tissues or cells to chemical exposure can be compared by determining the percentage of genes that were actually expressed. When the number of genes present on the array that were expressed in tissues or cells as determined by present/absent calls across all doses of RDX were compared, cell cultures were found to express a larger percentage of genes. Across all doses of RDX, 54% of the genes present on the array were expressed in liver, 62% of all possible genes on the array were expressed by primary hepatic cells at 24 hrs and 68% at 48 hrs of exposure to RDX. Primary cells at 24 hrs across all doses of RDX were more similar (88%) to overall liver tissue expression patterns than primary cells at 48 hrs (79%) when common expressed genes are examined. This data indicates that the cells are able to express many of the same genes expressed in liver tissues, a prerequisite for establishing similar responses.

**Figure 1:**
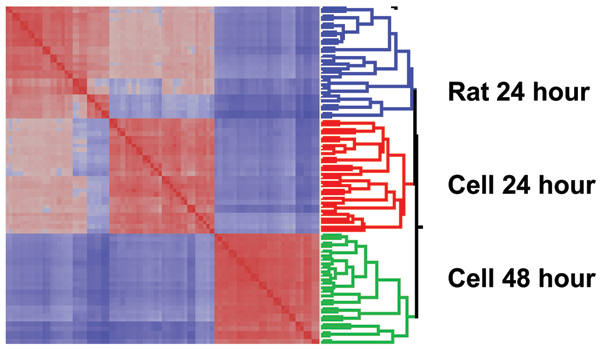
**Hierarchical clustering analysis of the correlation matrix of normalized data**. Blue squares represent low correlations and red represents high correlations.

**Figure 2:**
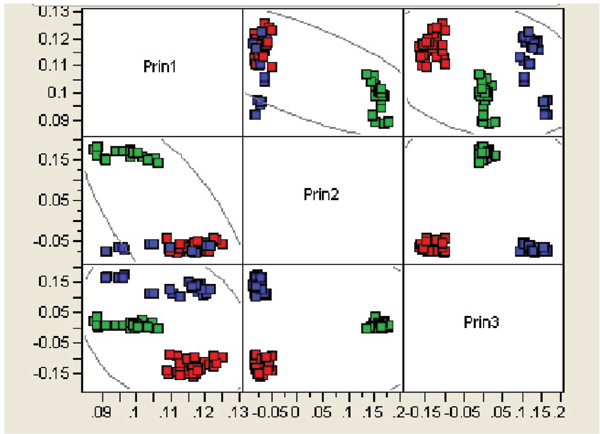
**Principal components analysis of relatedness between microarray data**. Each colored box represents an individual hybridization. Blue = liver tissue, Red = 24 hr exposed cells, and Green = 48 hr exposed cells. Prin1 = first principal component, Prin2 = second principal component, and Prin3 = third principal component.

**Figure 3:**
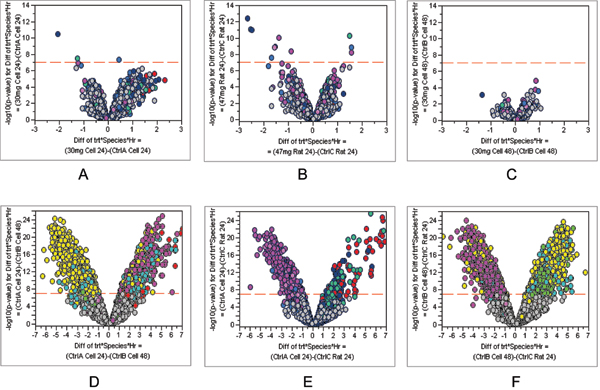
**Volcano plot illustrating predominant sources of differentially expressed genes**. Differentially expressed genes are highlighted according to a Bonferroni cutoff (red dashed line) between the high dose treatments and controls within the condition e.g. 24 hr cell (A), 48 hr cell (B) and rat (C), and between the controls of the three conditions ((24 hr cell – 48 hr cell (D), 24 hr cell -rat (E), and 48 hr cell -rat (F)).

**Figure 4:**
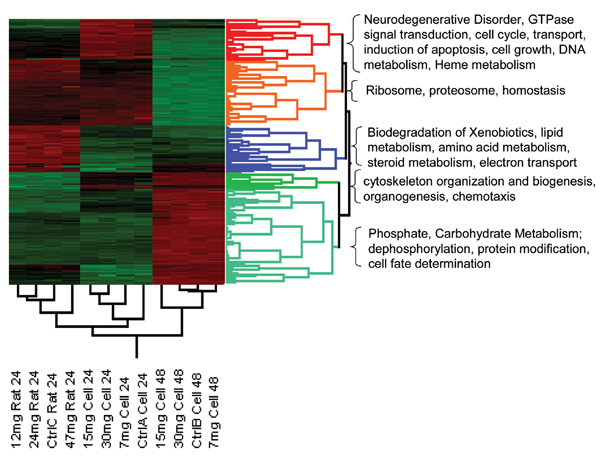
**Hierarchical clustering of significant differentially expressed genes according to Bonferroni correction, total 2929 genes**. Red represents up and green represents down-regulated genes relative to the mean of all samples. Cluster enriched GO terms are listed adjacent to each cluster.

Hierarchical clustering analysis of the correlation matrix of the normalized microarray ratio data shows clustering according to tissue and exposure type. The correlation of expression between and within samples suggests that tissues and cells are responding differently to RDX exposure (Figure 1). Principal components analysis of the same correlation matrix indicates that some similarity does exist between liver and cells exposed to RDX for 24 hrs (Figure 2). After fitting a mixed linear model to each gene, the resulting volcano plots (Figure 3) demonstrated that the numbers of significant differentially expressed genes (DEG) between the conditions (Figure 3D, 3E, 3F) were substantially more than the numbers of DEG due to the high dose treatment within the conditions (Figure 3A, 3B, 3C). This indicates that different conditions, including between liver tissue and cell cultures, and between cell culture conditions, had greater impact on the gene expression profiles than the treatment. Hierarchical clustering analysis of significant DEG with Bonferroni correction using ANOVA also indicates that 24 hr exposed cells are most similar to liver tissue with little overlap between treatments and cell timepoints (Figure 4).

**Figure 5:**
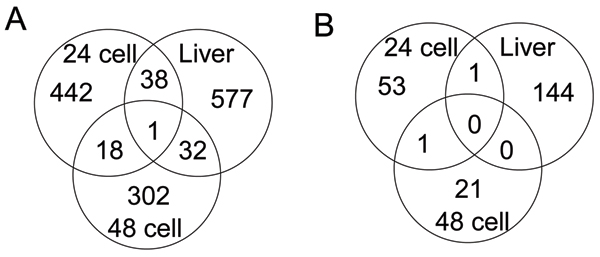
**Common differentially expressed genes among all doses of exposed primary hepatic cells and liver tissue determined by ANOVA**. Numbers in overlapping circles represent genes common to respective cells or tissue. 24 cell = 24 hr exposed cells, 48 cell = 48 hr exposed cells, and Liver = liver tissue. A. ANOVA analysis (p < 0.01). B. ANOVA analysis (p < 0.001).

We used the abundance of genes in common between differentially expressed gene lists to compare the responses of primary cell and liver tissue to RDX exposure (Figure 5). Depending on the significance level used to identify significant genes, up to 6.4% of the differentially expressed genes in liver tissue at all exposures were also differentially expressed in 24 hr primary cells at all exposures. Forty eight hr primary cells were less similar in differential expression to liver with up to 5.5% of genes differentially expressed in common with liver.

**Figure 6:**
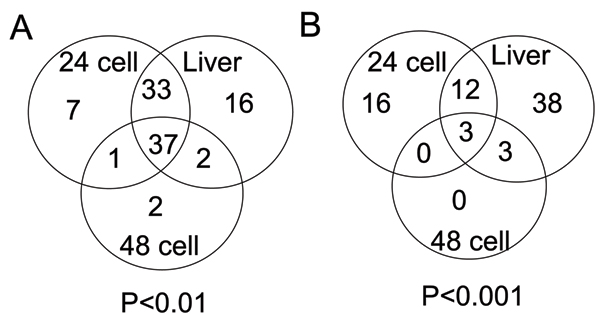
**Common KEGG pathway terms among all doses of exposed primary hepatic cells and liver tissue. Numbers represent pathways common to respective cells or tissue**. 24 cell = 24 hr exposed cells, 48 cell = 48 hr exposed cells, and Liver = liver tissue. A. ANOVA analysis (p < 0.01). B. ANOVA analysis (p < 0.001).

Analysis of differentially expressed genes held in common between conditions is a good indicator of relatedness. Functional biological pathways often consist of multiple genes. Therefore identification of shared pathways can be a more robust approach to comparing conditions. We compiled lists of pathways containing differentially expressed genes using EASE to identify pathways in the Kyoto Encycloaedia of Genes and Genomes (KEGG). Approximately 8.2% of affected genes had identifiable KEGG pathways. Twenty four hr exposed primary cells shared up to 78.4% of the pathways found to be affected in liver tissue (Figure 6). 48 hr exposed cells shared up to 44.3% of pathways with liver tissues.

The proteosome/protein and degradation KEGG pathway was identified as significantly enriched in treated liver tissues (EASE score 0.004). Glutathione metabolism and valine, leucine and isoleucine degradation were enriched in 24 hr treated cells (EASE scores 0.048 and 0.048). No significantly enriched pathways were detected in 48 hr treated cells. Liver and 24 hr treated cells were similarly enriched in GO terms such as main pathways of carbohydrate metabolism/energy derivation by oxidation of organic compounds, and macromolecule biosynthesis. Significant functional differences between primary cells and liver tissue were observed in enriched GO terms not held in common between conditions (Table 2).

Significantly changed genes in individual doses and exposure times of primary cells were compared to those found in different dose treatments of RDX. This comparison was performed to identify which dose and exposure time combination in primary cells was most similar to effects seen in liver tissue. Effects observed at 30 mg/L RDX for 24 hrs exposure had the highest number of genes in common with liver tissues at any dose of RDX.

## Discussion

We have compared the responses of an *in vitro *model system, primary hepatocyte cells, to that of livers in whole animals to determine the extent to which the model system faithfully represents *in vivo *effects. Primary hepatic cells expressed (as defined by presence absence calls) up to 88% of the same genes expressed by *in vivo *liver tissues when all control and RDX doses treatments were considered. The overlap in expressed genes indicates a significant conservation of global gene expression between the two systems. This is consistent with observations of others where primary hepatocyte cells maintained 80% similarity to liver tissue in global gene expression [8]. However comparison of expression in unexposed cells and tissue also illustrates significant functional differences that may exist between the different systems. The similarity of the *in vivo *and *in vitro *systems may be dependent upon length of time that the hepatocytes had been isolated from liver tissue rather than exposure time to toxicant. The similarity of basal gene expression in primary hepatocytes to that of liver has been shown to decrease as the isolated cells adapt to conditions in culture [8,9].

The overall similarity of responses of primary cells and liver tissue to RDX exposure was far less conserved than global presence and absence of expressed genes. Liver tissue responded differently to RDX than primary cells. Less than 6.4% of differentially expressed genes found in liver were also found in 24 hr or 48 hr primary cell exposures. This result is consistent with what Boess et al. [10] found, i.e. the limited overlap of significant DEGs from liver and two cell lines. However, the use of a p-value based rule for gene selection is not designed to maximize overlap; rather, it targets controlling the false positive rate in the final list. We used a conservative Bonferroni cutoff to provide strict control on the false positive rate. Even with this strict cutoff, almost 3000 genes were considered differentially expressed. Boess et al. [10] further cautioned about the comparison of the gene-to-gene basis between *in vitro *and *in vivo *systems. Studies comparing tumor tissues and tumor derived-cell lines have also indicated the considerable differences exist in gene expression between tissues and cell lines [11,12].

Biological functions enriched in differentially expressed gene lists highlight potentially major differences between primary cell physiology and liver tissue (Figure 4, Tables 1 and 2). General pathways and functions that were overrepresented in both liver tissue and 24 hr treated primary cells were involved in carbohydrate metabolism, and macromolecule biosynthesis. Enriched functions affected in 24 hr exposed primary cells that were not shared with liver were principally involved in cell growth, division, and proliferation (Figure 4). In 48 hr treated cells, protein degradation, fatty acid transport and transcriptional activity were enriched. In addition, several genes involved in apoptosis were differentially expressed although not significantly enriched in 48 hr treated cells. Liver tissues were enriched in pathways related to ubiquitin dependent protein catabolism, lipid and protein metabolism, oxido/reductase activity and transferase activity on nitrogen containing groups. These differences may be due to certain pathways being induced due to the trauma of cell isolation and adaptation to culturing conditions (cell proliferation, etc.), which overwhelms any response to the xenobiotics. Alternatively, it may a consequence of biotransformation-related genes whose expression are known to be down-regulated after cell isolation, such as P450s. This in turn can alter both the duration of exposure and the compounds to which the cells and/or liver are being exposed. Lastly, one cannot discount the potential involvement of other organ systems in the whole organism that are difficult to model with a single cell system. For example the toxicological activation of 2,4-dinitrotoluene has been shown to be a multi-step process involving metabolism in the liver, excretion into the bile, deconjugation of metabolites and further metabolism by the intestinal flora, re-uptake (enterohepatic transport) of metabolites into liver, and finally activation and binding to cellular macromolecules in the liver [13]. The absence of enrichment of functions related to metabolizing nitrogenous groups in 24 hr or 48 hr primary cells suggests that, at these time points, the hepatocyte cells reflect a limited range of the gene expression activities in liver tissue that respond to RDX.

## Conclusion

We have compared the similarity in gene expression responses of an *in vitro *system, primary hepatocyte cells, to an *in vivo *system, rat liver tissue, to determine how faithfully primary cells reflect toxicological responses of liver tissue exposed to an energetic compound, RDX. Primary cells were capable of expressing most genes present in liver tissue; however transcriptional level changes in primary cells reflected only a fraction of responses observed in liver tissue. This study indicates that care must be taken when toxicological data is derived from primary cells and extrapolated to whole animal organ toxicity effects.

## Methods

### Animal exposures

RDX (Purity > 99%) was obtained from Stan Caulder (Naval Surface Warfare Center, Indianhead, MD) and was stored under absolute ethanol. Female Sprague-Dawley rats were from the in-house breeding colony (School of Pharmacy, University of Louisiana at Monroe [ULM]) maintained in accordance with the Guide for Use and Care of Animals (National Academy of Science, 1996). Breeders were from Harlan-Sprague Dawley (Madison, WI). Rats were housed with free access to pelleted rodent chow (#7012, Harlan/Teklad, Madison, WI) and tap water and with a 12 hr light/dark cycle. One week prior to trials, groups of rats (175–225 gm) were housed individually in polycarbonate cages on hardwood bedding (Sani-chips, Harlan/Teklad, Madison, WI). Study protocols had prior approval by the ULM animal care and use committee.

Prior to treatment, food was withdrawn overnight. Five rats were randomly assigned to dose and exposed to single oral gavage doses of 0 (1:20, v/v, dimethylsulfoxide vehicle control in corn oil), 47, and 94 mg/Kg RDX. At 24 hrs post exposure animals were exsanguinated by cardiac puncture while under CO_2 _anesthesia, liver tissue samples harvested and immediately preserved in RNAlater (Ambion, Austin, TX). Concentrations of RDX in liver tissues were determined using the Environmental Protection Agency Method 8330 [14].

### Primary Hepatocyte Exposures

Primary rat hepatocyte cell suspensions were prepared from fresh primary isolates shipped the day of isolation (Cambrex, Rockland, ME). Cell viability was checked upon arrival (71 ± 8%), seeded into flasks (6 × 10^6 ^cells in a T75 flask) and allowed to settle for 24 hrs prior to dosing (dosing started at an estimated 36-hrs post isolation). Cells were dosed by exchanging the existing media with media spiked with a dimethylsulfoxide stock solution of the compound of interest (100 uL of dimethylsulfoxide solution into 10 mL media) or solvent alone for controls. Final media concentrations were 7.5, 15, or 30 mg/L. Cells were maintained in an incubator (37°C, 5% CO_2 _incubator, Nuaire, Plymouth, MN) in hepatocyte culture media and supplements as per supplier instructions (Cambrex Hepatocyte Culture Media, CC-3199; HCM singlequot supplements, CC-4182). After the 24 or 48 hr dose period, the cells were washed with phosphate buffered saline (GIBCO-InVitrogen, Grand Island, NY) and detached with 1 × trypsin-EDTA (GIBCO-InVitrogen, Grand Island, NY). The cells were left in tryspin-EDTA no longer than 5 minutes. Freshly warmed media was then added to neutralize the trypsin. The cells were then transferred into a tube and centrifuged. RNAlater was added to the cell pellet and stored at -20 C until genomic analysis.

For the cytotoxicity assay, 2 × 10^4 ^cells/well were plated in Type 1 Collagen-coated, 96-well plate (BD Biosciences, Palo Alto, CA). Cells were dosed as described above. At the end of the 24 or 48 hr exposure period, the medium from the plates was removed, and the cells washed with 1 × PBS. Freshly warmed Leibovitz medium (100 μl) was introduced to each well followed by Neutral Red Stain (10 μl). Leibovitz medium was selected because the medium has been shown not to interfere with the Neutral Red cytotoxicity assay (Sigma-Aldrich, In Vitro Toxicology Kit Tox-4). The plates were incubated again for 2 more hours, after which the medium was removed and the Solubilization Solution (200 μl) was added. The plates were placed on a shaker for 20 minutes to dissolve and mix any Neutral Red crystals formed. The plates were then read with a Tecan Safire (v 2.20 08/02) spectrophotometer at 540 nm and referenced at 690 nm.

### Microarray Hybridizations

**Figure 7:**
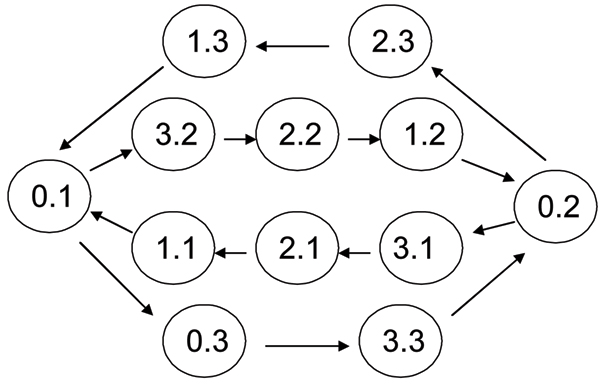
**Looped hybridization scheme for analysis of liver and hepatic cell transcriptional responses**. Circles represent treatment samples. For liver tissue samples, 0.x = solvent control biological replicate x, 1.x = 12 mg/kg dose biological replicate x, 2.x = 24 mg/kg dose biological replicate x, 3.x = 48 mg/kg dose biological replicate x. For 24 and 48 hr exposures of primary hepatic cells to RDX, 0.x = solvent control biological replicate x, 1.x = 7.5 mg/L dose biological replicate x, 2.x = 15 mg/L dose biological replicate x, 3.x = 30 mg/L dose biological replicate x. Arrows represent array hybridizations between respective samples where the arrowhead indicates dye labelling with Alexa 647 and the base of arrows indicate dye labelling with Cy3.

Total RNA was isolated from RNAeasy preserved livers and primary hepatic cells using Qiagen RNAmini kits (Valencia, CA). Total RNA from three biological replicates at each dose were compared using a loop design with dye swaps (Figure 7) [15]. cDNA from 1 ug total RNA was synthesized, hybridized to arrays, and detected by secondary hybridization to Alexa647 and Cy3 dendrimer oligonucleotides using an Array900 detection kit per manufacturer's instructions (Genisphere, Hatfield, PA). cDNA was hybridized to 8 K Sigma/Compugen rat 70-mer oligonucleotide libraries arrayed on glass slides (Center for Applied Genomics, Newark, NJ ). After secondary hybridization, slides were scanned using a 5 micron ChipReader microarray reader (BioRad, Hercules, CA). Spots were identified and quantified using VersArray software (BioRad).

### Gene expression analysis

Local background signals were subtracted from each spot signal using the mean pixel density of a ring around that spot which is 4 pixels wide. Net intensity of spots was cross-channel normalized using locally weighted linear regression procedure (Leoss). The spot signals were transformed to logarithmic (base 2) values. Loess smoothing was applied to normalize data twice: first, within the arrays to minimize the dye bias, and then across the arrays to normalize data across all runs. Spots were considered present if the net spot intensity was 2 standard deviations greater than the mean local background intensity. Mixed-model ANOVA was performed on the normalized intensities (not ratios) and resulting significance tests were used to identify differentially expression genes [16]. The aforementioned quality control, normalization, and statistical modelling were performed using JMP Genomics 2.0 from SAS Institute Inc. (Cary, NC. ). Pathway and gene ontology (GO) analyses for genes identified as differentially expressed by ANOVA were performed in ArrayTrack , and EASE [17]. We used the binomial test of Cho and Campbell [18] as implemented in PANTHER  to identify several classes of biological processes that were over or under represented in lists of these expressed genes compared to the array list that highlight potential functional differences in the tissue and cell types.

## List of abbreviations

DEG: Differentially Expressed Genes

EASE: Expression Analysis Systematic Explorer

GO: Gene Ontology

KEGG: Kyoto Encyclopedia of Genes and Genomes

RDX: hexahydro-1,3,5-trinitro-1,3,5-triazine

## Authors' contributions

EJP drafted the manuscript, contributed to the design of the study, and assisted with array analysis. WB conducted the statistical analysis. XG conducted the array hybridizations. CYA conducted the primary cell exposures. RDW contributed to the statistical analysis. TMC contributed to the statistical analysis. SAM conducted the rat exposures. LSI participated in the design of the study, assisted with the rat and cell exposures, and contributed to the writing of the manuscript. All authors read and approved the final manuscript.

## Figures and Tables

**Table 1:** Comparison classes of genes expressed in liver, 24 hr cells and 48 hr cells reveal significant differences between tissue and isolated cells.

**Biological Process**	**Liver**		**24 hr Cells**		**48 h Cells**	
	**over/under**	**P-value**	**over/under**	**P-value**	**over/under**	**P-value**
**Liver and Primary cells**						
Signal transduction	+	1.02E-21	+	3.71E-14	+	1.29E-10
Cell surface receptor mediated signal transduction	+	7.35E-12	+	1.16E-09	+	1.65E-07
Cell communication	+	9.37E-09	+	3.93E-06	+	4.89E-03
Neuronal activities	+	6.85E-08	+	3.36E-08	+	3.63E-05
G-protein mediated signaling	+	9.33E-07	+	1.42E-08	+	2.47E-06
Ion transport	+	8.51E-06	+	7.03E-09	+	1.34E-02
Cation transport	+	2.84E-05	+	1.52E-07	+	1.21E-02
Synaptic transmission	+	1.71E-04	+	8.40E-04	+	4.46E-02
Developmental processes	+	1.43E-05	+	1.71E-04	+	3.17E-01
Protein biosynthesis	-	1.68E-10	-	2.15E-09	-	1.00E+00
Cell adhesion	+	6.52E-03	+	4.22E-03	+	1.00E+00

**Liver only**						
Electron transport	-	4.11E-09	-	5.13E-02	-	6.33E-01
Lipid, fatty acid and steroid metabolism	-	4.38E-05	+	1.00E+00	-	1.00E+00
Steroid metabolism	-	1.56E-04	-	1.00E+00	-	1.00E+00
Ligand-mediated signaling	+	3.48E-04	+	1.07E-01	+	1.00E+00
Fatty acid metabolism	-	7.41E-04	-	1.00E+00	-	1.00E+00
Coenzyme and prosthetic group metabolism	-	1.45E-02	-	1.00E+00	+	1.00E+00
Oxidative phosphorylation	-	2.40E-02	-	7.57E-01	-	1.00E+00
Protein phosphorylation	+	3.10E-02	-	1.00E+00	-	1.00E+00
Cell adhesion-mediated signaling	+	3.80E-02	+	3.37E-01	+	1.00E+00

**Primary cells**						
Protein metabolism and modification	-	6.79E-02	-	2.47E-04	-	3.27E-03
Transport	+	6.85E-02	+	4.22E-06	+	1.33E-02
Protein folding	-	1.11E-01	-	1.94E-03	-	1.00E+00
General vesicle transport	-	1.00E+00	-	4.31E-02	-	1.00E+00
Intracellular protein traffic	-	1.00E+00	-	1.12E-03	-	1.00E+00
Nucleoside, nucleotide and nucleic acid metabolism	-	1.00E+00	-	1.68E-02	-	1.00E+00
Proteolysis	-	1.55E-01	-	1.00E+00	-	4.39E-02
Blood circulation and gas exchange	-	1.00E+00	+	6.59E-01	+	8.90E-03

Each list of genes present in liver or cells was compared to the reference list of all possible genes detectable by the microarray using the binomial test (18) for each molecular function, biological process, or pathway term in PANTHER . An over (+) or under (-) representation of PANTHER classification categories was considered significant when P-value < 0.05.

**Table 2:** Biological functions identified by GO term enrichment as uniquely affected by RDX in different treatments.

**Liver 24 hr after exposure**	**EASE score**	**24 hr exposure of primary hepatocyte cells**	**EASE score**	**48 hr exposure of primary hepatocyte cells**	**EASE score**
metabolism	9.00E-04	pathogenesis	2.30E-03	physiological process	3.75E-02
lipid metabolism	9.00E-04	lipid transport	1.78E-02	perception of abiotic stimulus	4.14E-02
fatty acid metabolism	5.50E-03	amino acid metabolism	2.77E-02	protein metabolism	4.27E-02
carboxylic acid metabolism	5.60E-03	energy derivation by oxidation of organic compounds	3.22E-02		
glutathione conjugation reaction	7.00E-03	cholesterol metabolism	3.33E-02		
ubiquitin-dependent protein catabolism	1.32E-02	amino acid and derivative metabolism	3.36E-02		
lipid biosynthesis	1.88E-02	RNA processing	3.90E-02		
sodium ion transport	3.47E-02				
blood pressure	4.00E-02				

Data sets were comprised of significantly changed genes as identified by ANOVA in all doses within a tissue or cell exposure time. An enrichment of terms was considered significant when EASE scores were less than 0.05 and a within system false discovery rate was less than 0.125.
